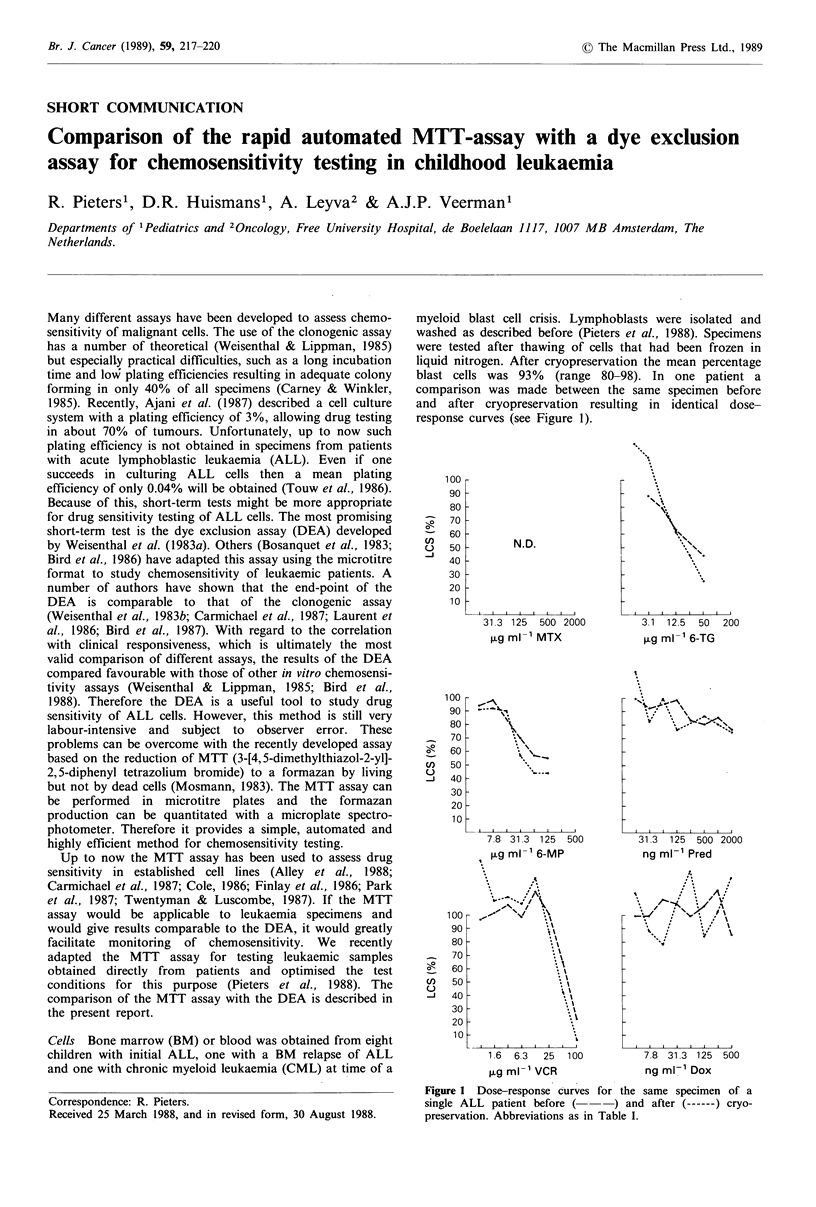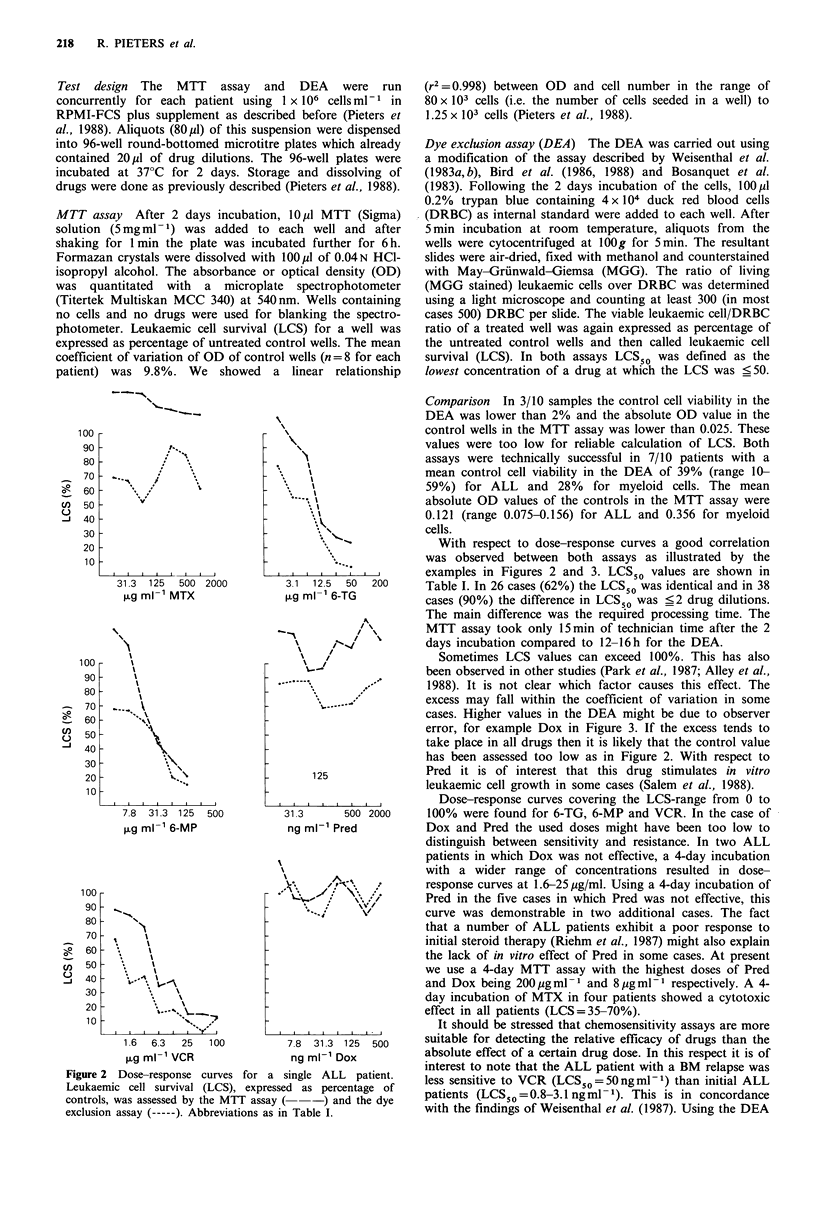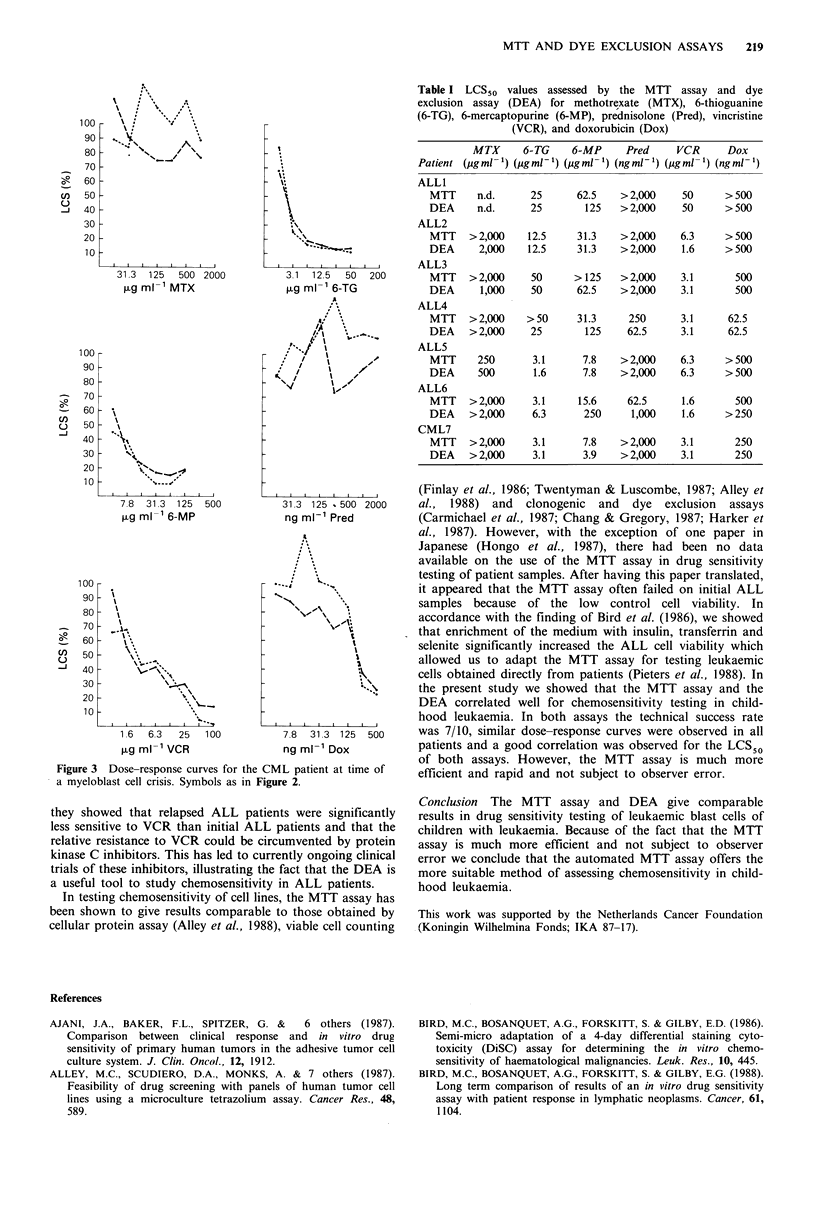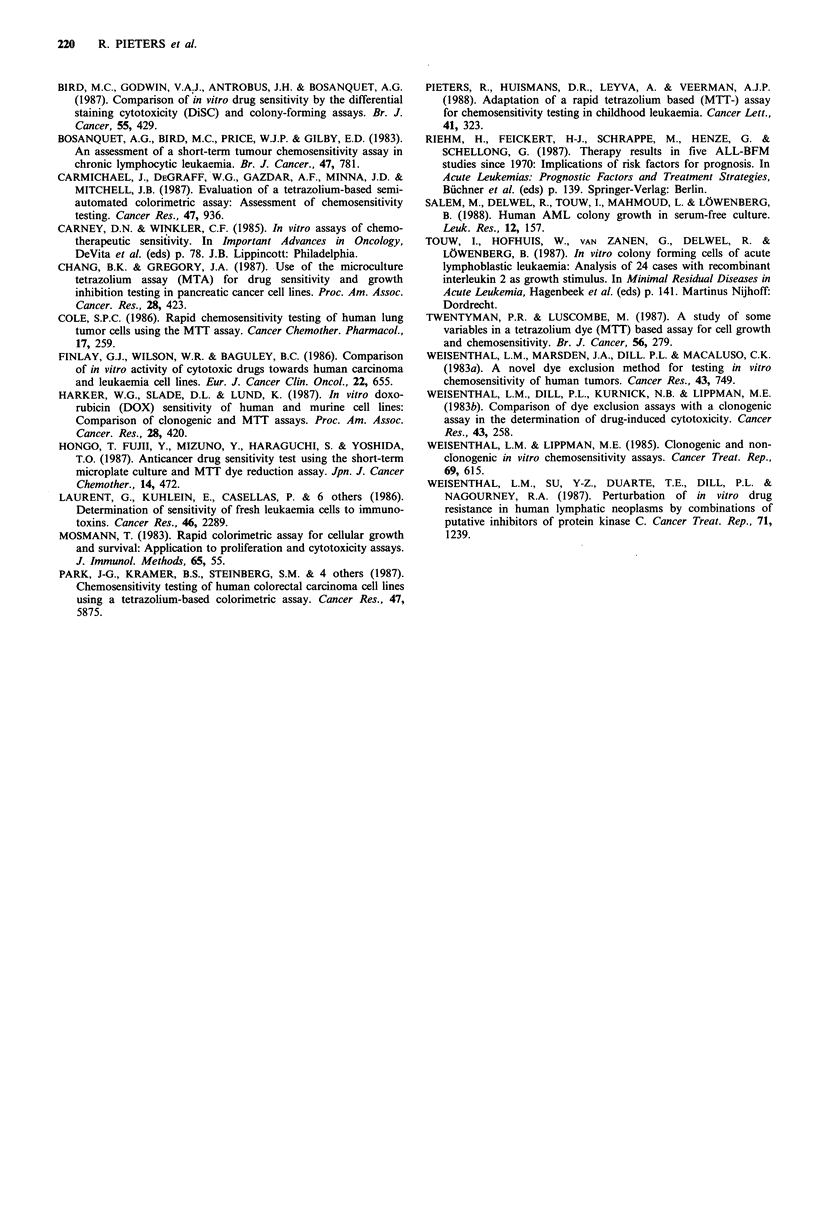# Comparison of the rapid automated MTT-assay with a dye exclusion assay for chemosensitivity testing in childhood leukaemia.

**DOI:** 10.1038/bjc.1989.44

**Published:** 1989-02

**Authors:** R. Pieters, D. R. Huismans, A. Leyva, A. J. Veerman

**Affiliations:** Department of Pediatrics, Free University Hospital, Amsterdam, The Netherlands.


*Br. J. Cancer* (1989), **59**, 217–220 © The Macmillan Press Ltd., 1989

SHORT COMMUNICATION

### Comparison of the rapid automated MTT-assay with a dye exclusion assay for chemosensitivity testing in childhood leukaemia

R. Pieters[1], D.R. Huismans[1], A. Leyva[2] & A.J.P. Veerman[1]

*Departments of [1]Pediatrics and [2]Oncology, Free University Hospital, de Boelelaan 1117, 1007 MB Amsterdam, The Netherlands.*

Many different assays have been developed to assess chemosensitivity of malignant cells. The use of the clonogenic assay has a number of theoretical (Weisenthal & Lippman, 1985) but especially practical difficulties, such as a long incubation time and low plating efficiencies resulting in adequate colony forming in only 40% of all specimens (Carney & Winkler, 1985). Recently, Ajani *et al.* (1987) described a cell culture system with a plating efficiency of 3%, allowing drug testing in about 70% of tumours. Unfortunately, up to now such plating efficiency is not obtained in specimens from patients with acute lymphoblastic leukaemia (ALL). Even if one succeeds in culturing ALL cells then a mean plating efficiency of only 0.04% will be obtained (Touw *et al.*, 1986). Because of this, short-term tests might be more appropriate for drug sensitivity testing of ALL cells. The most promising short-term test is the dye exclusion assay (DEA) developed by Weisenthal *et al.* (1983*a*). Others (Bosanquet *et al.*, 1983; Bird *et al.*, 1986) have adapted this assay using the microtitre format to study chemosensitivity of leukaemic patients. A number of authors have shown that the end-point of the DEA is comparable to that of the clonogenic assay (Weisenthal *et al.*, 1983*b*; Carmichael *et al.*, 1987; Laurent *et al.*, 1986; Bird *et al.*, 1987). With regard to the correlation with clinical responsiveness, which is ultimately the most valid comparison of different assays, the results of the DEA compared favourable with those of other *in vitro* chemosensitivity assays (Weisenthal & Lippman, 1985; Bird *et al.*, 1988). Therefore the DEA is a useful tool to study drug sensitivity of ALL cells. However, this method is still very labour-intensive and subject to observer error. These problems can be overcome with the recently developed assay based on the reduction of MTT (3-[4,5-dimethylthiazol-2-yl]-2,5-diphenyl tetrazolium bromide) to a formazan by living but not by dead cells (Mosmann, 1983). The MTT assay can be performed in microtitre plates and the formazan production can be quantitated with a microplate spectrophotometer. Therefore it provides a simple, automated and highly efficient method for chemosensitivity testing.

Up to now the MTT assay has been used to assess drug sensitivity in established cell lines (Alley *et al.*, 1988; Carmichael *et al.*, 1987; Cole, 1986; Finlay *et al.*, 1986; Park *et al.*, 1987; Twentyman & Luscombe, 1987). If the MTT assay would be applicable to leukaemia specimens and would give results comparable to the DEA, it would greatly facilitate monitoring of chemosensitivity. We recently adapted the MTT assay for testing leukaemic samples obtained directly from patients and optimised the test conditions for this purpose (Pieters *et al.*, 1988). The comparison of the MTT assay with the DEA is described in the present report.

*Cells* Bone marrow (BM) or blood was obtained from eight children with initial ALL, one with a BM relapse of ALL and one with chronic myeloid leukaemia (CML) at time of a myeloid blast cell crisis. Lymphoblasts were isolated and washed as described before (Pieters *et al.*, 1988). Specimens were tested after thawing of cells that had been frozen in liquid nitrogen. After cryopreservation the mean percentage blast cells was 93% (range 80–98). In one patient a comparison was made between the same specimen before and after cryopreservation resulting in identical dose–response curves (see Figure 1).

**Figure 1** Dose–response curves for the same specimen of a single ALL patient before (———) and after (------) cryopreservation. Abbreviations as in Table I.

Correspondence: R. Pieters.
Received 25 March 1988, and in revised form, 30 August 1988.

*Test design* The MTT assay and DEA were run concurrently for each patient using $1 \times 10^6$ cells $ml^{-1}$ in RPMI-FCS plus supplement as described before (Pieters *et al.*, 1988). Aliquots (80 µl) of this suspension were dispensed into 96-well round-bottomed microtitre plates which already contained 20 µl of drug dilutions. The 96-well plates were incubated at 37°C for 2 days. Storage and dissolving of drugs were done as previously described (Pieters *et al.*, 1988).

*MTT assay* After 2 days incubation, 10 µl MTT (Sigma) solution (5 mg $ml^{-1}$) was added to each well and after shaking for 1 min the plate was incubated further for 6 h. Formazan crystals were dissolved with 100 µl of 0.04 N HCl-isopropyl alcohol. The absorbance or optical density (OD) was quantitated with a microplate spectrophotometer (Titertek Multiskan MCC 340) at 540 nm. Wells containing no cells and no drugs were used for blanking the spectrophotometer. Leukaemic cell survival (LCS) for a well was expressed as percentage of untreated control wells. The mean coefficient of variation of OD of control wells ($n = 8$ for each patient) was 9.8%. We showed a linear relationship ($r^2 = 0.998$) between OD and cell number in the range of $80 \times 10^3$ cells (i.e. the number of cells seeded in a well) to $1.25 \times 10^3$ cells (Pieters *et al.*, 1988).

**Figure 2** Dose–response curves for a single ALL patient. Leukaemic cell survival (LCS), expressed as percentage of controls, was assessed by the MTT assay (———) and the dye exclusion assay (-----). Abbreviations as in Table I.

*Dye exclusion assay (DEA)* The DEA was carried out using a modification of the assay described by Weisenthal *et al.* (1983*a*, *b*), Bird *et al.* (1986, 1988) and Bosanquet *et al.* (1983). Following the 2 days incubation of the cells, 100 µl 0.2% trypan blue containing $4 \times 10^4$ duck red blood cells (DRBC) as internal standard were added to each well. After 5 min incubation at room temperature, aliquots from the wells were cytocentrifuged at 100 *g* for 5 min. The resultant slides were air-dried, fixed with methanol and counterstained with May–Grünwald–Giemsa (MGG). The ratio of living (MGG stained) leukaemic cells over DRBC was determined using a light microscope and counting at least 300 (in most cases 500) DRBC per slide. The viable leukaemic cell/DRBC ratio of a treated well was again expressed as percentage of the untreated control wells and then called leukaemic cell survival (LCS). In both assays $LCS_{50}$ was defined as the *lowest* concentration of a drug at which the LCS was $\leq 50$.

*Comparison* In 3/10 samples the control cell viability in the DEA was lower than 2% and the absolute OD value in the control wells in the MTT assay was lower than 0.025. These values were too low for reliable calculation of LCS. Both assays were technically successful in 7/10 patients with a mean control cell viability in the DEA of 39% (range 10–59%) for ALL and 28% for myeloid cells. The mean absolute OD values of the controls in the MTT assay were 0.121 (range 0.075–0.156) for ALL and 0.356 for myeloid cells.

With respect to dose–response curves a good correlation was observed between both assays as illustrated by the examples in Figures 2 and 3. $LCS_{50}$ values are shown in Table I. In 26 cases (62%) the $LCS_{50}$ was identical and in 38 cases (90%) the difference in $LCS_{50}$ was $\leq 2$ drug dilutions. The main difference was the required processing time. The MTT assay took only 15 min of technician time after the 2 days incubation compared to 12–16 h for the DEA.

Sometimes LCS values can exceed 100%. This has also been observed in other studies (Park *et al.*, 1987; Alley *et al.*, 1988). It is not clear which factor causes this effect. The excess may fall within the coefficient of variation in some cases. Higher values in the DEA might be due to observer error, for example Dox in Figure 3. If the excess tends to take place in all drugs then it is likely that the control value has been assessed too low as in Figure 2. With respect to Pred it is of interest that this drug stimulates *in vitro* leukaemic cell growth in some cases (Salem *et al.*, 1988).

Dose–response curves covering the LCS-range from 0 to 100% were found for 6-TG, 6-MP and VCR. In the case of Dox and Pred the used doses might have been too low to distinguish between sensitivity and resistance. In two ALL patients in which Dox was not effective, a 4-day incubation with a wider range of concentrations resulted in dose–response curves at 1.6–25 µg/ml. Using a 4-day incubation of Pred in the five cases in which Pred was not effective, this curve was demonstrable in two additional cases. The fact that a number of ALL patients exhibit a poor response to initial steroid therapy (Riehm *et al.*, 1987) might also explain the lack of *in vitro* effect of Pred in some cases. At present we use a 4-day MTT assay with the highest doses of Pred and Dox being 200 µg $ml^{-1}$ and 8 µg $ml^{-1}$ respectively. A 4-day incubation of MTX in four patients showed a cytotoxic effect in all patients (LCS = 35–70%).

It should be stressed that chemosensitivity assays are more suitable for detecting the relative efficacy of drugs than the absolute effect of a certain drug dose. In this respect it is of interest to note that the ALL patient with a BM relapse was less sensitive to VCR ($LCS_{50} = 50$ ng $ml^{-1}$) than initial ALL patients ($LCS_{50} = 0.8$–3.1 ng $ml^{-1}$). This is in concordance with the findings of Weisenthal *et al.* (1987). Using the DEA

**Figure 3** Dose–response curves for the CML patient at time of a myeloblast cell crisis. Symbols as in **Figure 2**.

they showed that relapsed ALL patients were significantly less sensitive to VCR than initial ALL patients and that the relative resistance to VCR could be circumvented by protein kinase C inhibitors. This has led to currently ongoing clinical trials of these inhibitors, illustrating the fact that the DEA is a useful tool to study chemosensitivity in ALL patients.

In testing chemosensitivity of cell lines, the MTT assay has been shown to give results comparable to those obtained by cellular protein assay (Alley *et al.*, 1988), viable cell counting

**Table I** $LCS_{50}$ values assessed by the MTT assay and dye exclusion assay (DEA) for methotrexate (MTX), 6-thioguanine (6-TG), 6-mercaptopurine (6-MP), prednisolone (Pred), vincristine (VCR), and doxorubicin (Dox)

| *Patient* | *MTX* ($\mu g\,ml^{-1}$) | *6-TG* ($\mu g\,ml^{-1}$) | *6-MP* ($\mu g\,ml^{-1}$) | *Pred* ($ng\,ml^{-1}$) | *VCR* ($\mu g\,ml^{-1}$) | *Dox* ($ng\,ml^{-1}$) |
|---|---|---|---|---|---|---|
| ALL1 | | | | | | |
| MTT | n.d. | 25 | 62.5 | >2,000 | 50 | >500 |
| DEA | n.d. | 25 | 125 | >2,000 | 50 | >500 |
| ALL2 | | | | | | |
| MTT | >2,000 | 12.5 | 31.3 | >2,000 | 6.3 | >500 |
| DEA | 2,000 | 12.5 | 31.3 | >2,000 | 1.6 | >500 |
| ALL3 | | | | | | |
| MTT | >2,000 | 50 | >125 | >2,000 | 3.1 | 500 |
| DEA | 1,000 | 50 | 62.5 | >2,000 | 3.1 | 500 |
| ALL4 | | | | | | |
| MTT | >2,000 | >50 | 31.3 | 250 | 3.1 | 62.5 |
| DEA | >2,000 | 25 | 125 | 62.5 | 3.1 | 62.5 |
| ALL5 | | | | | | |
| MTT | 250 | 3.1 | 7.8 | >2,000 | 6.3 | >500 |
| DEA | 500 | 1.6 | 7.8 | >2,000 | 6.3 | >500 |
| ALL6 | | | | | | |
| MTT | >2,000 | 3.1 | 15.6 | 62.5 | 1.6 | 500 |
| DEA | >2,000 | 6.3 | 250 | 1,000 | 1.6 | >250 |
| CML7 | | | | | | |
| MTT | >2,000 | 3.1 | 7.8 | >2,000 | 3.1 | 250 |
| DEA | >2,000 | 3.1 | 3.9 | >2,000 | 3.1 | 250 |

(Finlay *et al.*, 1986; Twentyman & Luscombe, 1987; Alley *et al.*, 1988) and clonogenic and dye exclusion assays (Carmichael *et al.*, 1987; Chang & Gregory, 1987; Harker *et al.*, 1987). However, with the exception of one paper in Japanese (Hongo *et al.*, 1987), there had been no data available on the use of the MTT assay in drug sensitivity testing of patient samples. After having this paper translated, it appeared that the MTT assay often failed on initial ALL samples because of the low control cell viability. In accordance with the finding of Bird *et al.* (1986), we showed that enrichment of the medium with insulin, transferrin and selenite significantly increased the ALL cell viability which allowed us to adapt the MTT assay for testing leukaemic cells obtained directly from patients (Pieters *et al.*, 1988). In the present study we showed that the MTT assay and the DEA correlated well for chemosensitivity testing in childhood leukaemia. In both assays the technical success rate was 7/10, similar dose–response curves were observed in all patients and a good correlation was observed for the $LCS_{50}$ of both assays. However, the MTT assay is much more efficient and rapid and not subject to observer error.

*Conclusion* The MTT assay and DEA give comparable results in drug sensitivity testing of leukaemic blast cells of children with leukaemia. Because of the fact that the MTT assay is much more efficient and not subject to observer error we conclude that the automated MTT assay offers the more suitable method of assessing chemosensitivity in childhood leukaemia.

This work was supported by the Netherlands Cancer Foundation (Koningin Wilhelmina Fonds; IKA 87–17).

### References

AJANI, J.A., BAKER, F.L., SPITZER, G. & 6 others (1987). Comparison between clinical response and *in vitro* drug sensitivity of primary human tumors in the adhesive tumor cell culture system. *J. Clin. Oncol.*, **12,** 1912.

ALLEY, M.C., SCUDIERO, D.A., MONKS, A. & 7 others (1987). Feasibility of drug screening with panels of human tumor cell lines using a microculture tetrazolium assay. *Cancer Res.*, **48,** 589.

BIRD, M.C., BOSANQUET, A.G., FORSKITT, S. & GILBY, E.D. (1986). Semi-micro adaptation of a 4-day differential staining cytotoxicity (DiSC) assay for determining the *in vitro* chemosensitivity of haematological malignancies. *Leuk. Res.*, **10,** 445.

BIRD, M.C., BOSANQUET, A.G., FORSKITT, S. & GILBY, E.G. (1988). Long term comparison of results of an *in vitro* drug sensitivity assay with patient response in lymphatic neoplasms. *Cancer*, **61,** 1104.

BIRD, M.C., GODWIN, V.A.J., ANTROBUS, J.H. & BOSANQUET, A.G. (1987). Comparison of *in vitro* drug sensitivity by the differential staining cytotoxicity (DiSC) and colony-forming assays. *Br. J. Cancer*, **55**, 429.

BOSANQUET, A.G., BIRD, M.C., PRICE, W.J.P. & GILBY, E.D. (1983). An assessment of a short-term tumour chemosensitivity assay in chronic lymphocytic leukaemia. *Br. J. Cancer.*, **47**, 781.

CARMICHAEL, J., DeGRAFF, W.G., GAZDAR, A.F., MINNA, J.D. & MITCHELL, J.B. (1987). Evaluation of a tetrazolium-based semiautomated colorimetric assay: Assessment of chemosensitivity testing. *Cancer Res.*, **47**, 936.

CARNEY, D.N. & WINKLER, C.F. (1985). *In vitro* assays of chemotherapeutic sensitivity. In *Important Advances in Oncology*, DeVita *et al.* (eds) p. 78. J.B. Lippincott: Philadelphia.

CHANG, B.K. & GREGORY, J.A. (1987). Use of the microculture tetrazolium assay (MTA) for drug sensitivity and growth inhibition testing in pancreatic cancer cell lines. *Proc. Am. Assoc. Cancer. Res.*, **28**, 423.

COLE, S.P.C. (1986). Rapid chemosensitivity testing of human lung tumor cells using the MTT assay. *Cancer Chemother. Pharmacol.*, **17**, 259.

FINLAY, G.J., WILSON, W.R. & BAGULEY, B.C. (1986). Comparison of *in vitro* activity of cytotoxic drugs towards human carcinoma and leukaemia cell lines. *Eur. J. Cancer Clin. Oncol.*, **22**, 655.

HARKER, W.G., SLADE, D.L. & LUND, K. (1987). *In vitro* doxorubicin (DOX) sensitivity of human and murine cell lines: Comparison of clonogenic and MTT assays. *Proc. Am. Assoc. Cancer. Res.*, **28**, 420.

HONGO, T. FUJII, Y., MIZUNO, Y., HARAGUCHI, S. & YOSHIDA, T.O. (1987). Anticancer drug sensitivity test using the short-term microplate culture and MTT dye reduction assay. *Jpn. J. Cancer Chemother.*, **14**, 472.

LAURENT, G., KUHLEIN, E., CASELLAS, P. & 6 others (1986). Determination of sensitivity of fresh leukaemia cells to immunotoxins. *Cancer Res.*, **46**, 2289.

MOSMANN, T. (1983). Rapid colorimetric assay for cellular growth and survival: Application to proliferation and cytotoxicity assays. *J. Immunol. Methods*, **65**, 55.

PARK, J-G., KRAMER, B.S., STEINBERG, S.M. & 4 others (1987). Chemosensitivity testing of human colorectal carcinoma cell lines using a tetrazolium-based colorimetric assay. *Cancer Res.*, **47**, 5875.

PIETERS, R., HUISMANS, D.R., LEYVA, A. & VEERMAN, A.J.P. (1988). Adaptation of a rapid tetrazolium based (MTT-) assay for chemosensitivity testing in childhood leukaemia. *Cancer Lett.*, **41**, 323.

RIEHM, H., FEICKERT, H-J., SCHRAPPE, M., HENZE, G. & SCHELLONG, G. (1987). Therapy results in five ALL-BFM studies since 1970: Implications of risk factors for prognosis. In *Acute Leukemias: Prognostic Factors and Treatment Strategies*, Büchner *et al.* (eds) p. 139. Springer-Verlag: Berlin.

SALEM, M., DELWEL, R., TOUW, I., MAHMOUD, L. & LÖWENBERG, B. (1988). Human AML colony growth in serum-free culture. *Leuk. Res.*, **12**, 157.

TOUW, I., HOFHUIS, W., VAN ZANEN, G., DELWEL, R. & LÖWENBERG, B. (1987). *In vitro* colony forming cells of acute lymphoblastic leukaemia: Analysis of 24 cases with recombinant interleukin 2 as growth stimulus. In *Minimal Residual Diseases in Acute Leukemia*, Hagenbeek *et al.* (eds) p. 141. Martinus Nijhoff: Dordrecht.

TWENTYMAN, P.R. & LUSCOMBE, M. (1987). A study of some variables in a tetrazolium dye (MTT) based assay for cell growth and chemosensitivity. *Br. J. Cancer*, **56**, 279.

WEISENTHAL, L.M., MARSDEN, J.A., DILL. P.L. & MACALUSO, C.K. (1983*a*). A novel dye exclusion method for testing *in vitro* chemosensitivity of human tumors. *Cancer Res.*, **43**, 749.

WEISENTHAL, L.M., DILL, P.L., KURNICK, N.B. & LIPPMAN, M.E. (1983*b*). Comparison of dye exclusion assays with a clonogenic assay in the determination of drug-induced cytotoxicity. *Cancer Res.*, **43**, 258.

WEISENTHAL, L.M. & LIPPMAN, M.E. (1985). Clonogenic and nonclonogenic *in vitro* chemosensitivity assays. *Cancer Treat. Rep.*, **69**, 615.

WEISENTHAL, L.M., SU, Y-Z., DUARTE, T.E., DILL, P.L. & NAGOURNEY, R.A. (1987). Perturbation of *in vitro* drug resistance in human lymphatic neoplasms by combinations of putative inhibitors of protein kinase C. *Cancer Treat. Rep.*, **71**, 1239.